# Mitochondrial Reverse Electron Transport: Mechanisms, Pathophysiological Roles, and Therapeutic Potential

**DOI:** 10.3390/biology14091140

**Published:** 2025-08-29

**Authors:** Yanyu Bao, Cuilan Hu, Bing Wang, Xiongxiong Liu, Qingfeng Wu, Dan Xu, Zheng Shi, Chao Sun

**Affiliations:** 1 Institute of Modern Physics, Chinese Academy of Sciences, Lanzhou 730000, China; baoyanyu@impcas.ac.cn (Y.B.); hucuilan@impcas.ac.cn (C.H.); lxx002@impcas.ac.cn (X.L.); wuqf@impcas.ac.cn (Q.W.); xudan@impcas.ac.cn (D.X.); 2 Key Laboratory of Heavy Ion Radiation Biology and Medicine, Chinese Academy of Sciences, Lanzhou 730000, China; 3 Key Laboratory of Basic Research on Heavy Ion Radiation Application in Medicine, Lanzhou 730000, China; 4 University of Chinese Academy of Sciences, Beijing 100049, China; 5 Institute for Radiological Science, National Institutes for Quantum Science and Technology (QST), Chiba 263-8555, Japan; wang.bing@qst.go.jp; 6 School of Biopharmaceutical and Engineering, Lanzhou Jiaotong University, Lanzhou 730070, China; shizheng19930326@163.com

**Keywords:** mitochondrial reverse electron transport, ischemia–reperfusion, neurodegenerative diseases, cancer, tuberculosis

## Abstract

Mitochondrial reverse electron transport (RET) represents a fundamental but potentially hazardous metabolic process in eukaryotic cells. This review systematically examines current understanding of RET mechanisms and their pathophysiological consequences. RET occurs when electrons flow inversely from reduced coenzyme Q (CoQH_2_) to complex I, driven by excessive reduction of the CoQ pool and elevated mitochondrial membrane potential, resulting in substantial superoxide production. While moderate RET contributes to physiological redox signaling, sustained RET activation leads to oxidative damage and activates regulated cell death pathways. Notably, RET demonstrates metabolic duality: it facilitates ATP generation through NAD^+^ reduction while simultaneously inducing mitochondrial dysfunction via reactive oxygen species overproduction. Pathologically, RET has been implicated in myocardial ischemia–reperfusion injury, neurodegenerative disorders including Alzheimer’s diseases, and exhibits context-dependent roles in tumor progression. Emerging evidence also suggests RET involvement in microbial pathogenesis through modulation of host immune responses. These findings position RET as a critical regulatory node in cellular metabolism with broad implications for human diseases. Future investigations should focus on developing tissue-specific RET modulators and elucidating the molecular switches governing its activation threshold, which may yield novel therapeutic strategies for diverse pathological conditions.

## Simple Summary

Mitochondria normally produce energy through an orderly electron transfer process along the respiratory chain. However, under certain conditions like excess succinate accumulation, electrons can flow backward in a phenomenon called reverse electron transport (RET). This review comprehensively examines RET from multiple perspectives: First, we explain its molecular mechanisms—how high mitochondrial membrane potential and reduced coenzyme Q pool drive electrons to reverse through complex I, generating reactive oxygen species (ROS). Second, we discuss RET’s dual biological roles: while moderate ROS serve as signaling molecules, excessive RET causes oxidative damage. Importantly, we analyze RET’s involvement in diverse physiological processes including immune response regulation, metabolic adaptation, and cell death pathways like ferroptosis and necroptosis. The review then details RET’s pathological impacts across multiple diseases: exacerbating tissue damage in heart attacks and strokes, contributing to neurodegeneration in Alzheimer’s disease, playing complex roles in cancer progression, and influencing host–pathogen interactions in tuberculosis. By systematically integrating current research, we provide a unified framework for understanding RET’s multifaceted nature—from its fundamental biochemical principles to its broad physiological and pathological consequences. This synthesis not only advances basic knowledge of mitochondrial biology but also highlights potential therapeutic strategies targeting RET in various diseases.

## 1. Introduction

Mitochondria, often referred to as the “powerhouses” of the cell, are double-membraned organelles that play a central role in energy metabolism. Typically exhibiting a rod-like or granular morphology, mitochondria are structurally compartmentalized into four distinct regions: the outer mitochondrial membrane, the intermembrane space, the inner mitochondrial membrane, and the matrix. Each compartment harbors specific enzymes, transporters, and biomolecules essential for mitochondrial functions [1], including metabolite exchange, redox reactions, and energy conversion processes [2]. The inner membrane, in particular, houses the protein complexes responsible for oxidative phosphorylation, the process by which the majority of cellular adenosine triphosphate (ATP) is generated, thereby positioning mitochondria at the core of cellular energy metabolism.

At the core of mitochondrial energy production is the ETC, also known as the respiratory chain (RC), which comprises a series of protein complexes (I–IV) embedded in the inner mitochondrial membrane of eukaryotes or in the plasma membrane of prokaryotes. These complexes mediate the sequential transfer of electrons from specific donors: complex I receives electrons from NADH, while complex II (succinate dehydrogenase) obtains electrons from succinate, with FADH_2_ functioning as an internal electron carrier within complex II during this process [3].

While oxidative phosphorylation is indispensable for energy generation, it is also a significant source of reactive oxygen species (ROS). ROS constitute a group of partially reduced oxygen derivatives, including superoxide anion (O_2_^•−^), hydroxyl radicals (•OH), peroxyl radicals (ROO•), alkoxyl radicals (RO•), and hydrogen peroxide radicals (H_2_O_2_) [4,5]. Historically, ROS were considered harmful by-products of aerobic metabolism, implicated in oxidative damage during ischaemia–reperfusion (I/R) injury, neurodegenerative diseases such as Alzheimer’s and the aging process [6]. However, emerging evidence reveals that mitochondrial ROS (mtROS) also function as essential signalling molecules that modulate various physiological processes, including cell proliferation, differentiation, immune responses, and apoptosis [7].

An important mechanism underlying mitochondrial ROS production is RET, a phenomenon first described by Chance and Hollunger in 1961 [8]. RET occurs when electrons flow in the reverse direction through complex I, typically under conditions of high membrane potential and an excessively reduced coenzyme Q (CoQ). This situation often arises when succinate, a substrate for complex II, is abundant. Under such conditions, electrons transferred from succinate reduce ubiquinone to ubiquinol, which then donates electrons back to complex I, reducing NAD^+^ to NADH. This reverse flux through complex I is associated with elevated ROS production, particularly at the flavin mononucleotide site of complex I [9]. RET-generated ROS have been implicated in both physiological and pathological contexts, including hypoxic adaptation, innate immune responses, and ischemic preconditioning. Understanding the regulation and consequences of RET is therefore critical for elucidating the complex roles of mitochondrial redox signaling in health and disease.

## 2. Mitochondrial Electron Transport Chain

### 2.1. Physiological Function of the Electron Transport Chain

Eukaryotic mitochondria are bounded by a pair of lipid bilayers and possess their own genetic machinery; within their matrix and cristae, substrate oxidation is coupled to a chemiosmotic circuit that converts electrochemical potential into the ATP currency required for cellular work and whole-body homeostasis. This process occurs on the inner mitochondrial membrane and is mediated by the electron transport chain (ETC)—an enzymatic system that transfers electrons from NADH and FADH_2_ to oxygen while pumping protons to form a gradient, thereby driving ATP synthesis [10]. As a core component of cellular metabolism, the ETC plays a pivotal role in energy conversion and the maintenance of normal physiological functions. Its efficiency is critical for cellular homeostasis, facilitating adaptation to fluctuating energy demands and regulating mitochondrial oxidative phosphorylation to reshape energy metabolism, which in turn influences cell growth and differentiation [11].

Beyond its role in energy metabolism, the ETC also exerts significant effects on immunometabolism. Studies have demonstrated that remodeling of the mitochondrial ETC involves alterations in the activity of ETC complexes (I-V), modulations in their interaction modes, or shifts in the balance between electron transport efficiency, energy output (e.g., ATP), and the production of signaling molecules (e.g., ROS). For instance, in adaptive immunity, the ETC regulates the functions of T and B cells: complex I sustains T helper (Th) cell proliferation; complex II modulates Th1 proliferation and cytokine production (e.g., IFN-γ); complex III is essential for the inhibitory function of Tregs; and Complex V supports Th17 differentiation. OXPHOS serves as the basis for IL-10 production by Bregs. Thus, the ETC can regulate multiple immune processes in mammals and promote the effector and regulatory functions of T and B lymphocytes [12]. Abnormal ETC function is closely linked to the pathogenesis of various diseases: in Alzheimer’s disease (AD), for example, ETC impairment induces neuronal apoptosis through ROS-mediated neuroinflammation (immune response) and cytochrome c-dependent apoptotic signaling (neuronal apoptotic signaling) [13,14].

Additionally, the ETC exquisitely regulates apoptosis, hypoxic adaptation, tumor immunity, athletic performance, and fertility through multiple molecular pathways. In the apoptotic pathway, the reversible phosphorylation state of cytochrome c dictates its release into the cytoplasm, thereby precisely controlling the caspase cascade [15]. In hypoxic adaptation mechanisms, the electron transport efficiency of complex I can be directly restored independently of the HIF pathway, ensuring ATP production [16]. Within the tumor immune microenvironment, inhibition of complex II leads to succinate accumulation, which inhibits histone demethylases and upregulates MHC-I antigen presentation, thereby enhancing CD8^+^ T cell-mediated antitumor effects [17]. In exercise physiology, synergistic enhancement of ETC flux and OXPHOS coupling efficiency can markedly increase skeletal muscle ATP production within hours, improving power output and endurance performance [18]. In male reproductive biology, reduced complex III activity significantly impairs bovine sperm motility parameters, indicating that mitochondrial OXPHOS plays a critical role in sperm quality and fertility potential [19]. Regarding inflammatory responses, inhibitors of complexes I, II, III, and V can block NLRP3 inflammasome activation, a process dependent on cytoplasmic ATP levels maintained by the mitochondrial ATP-phosphocreatine (PCr) shuttle rather than ROS [20]. During adaptive thermogenesis, cold exposure induces an approximate 25° rotation of the CIII_2_ supercomplex in brown adipose tissue mitochondria (adopting a type 2 conformation), shortening the CoQ exchange distance between complexes and enhancing electron transport efficiency. Coupled with membrane lipid remodeling (increased PE/PC ratio) and activation of the PERK-mediated lipid synthesis pathway, this conformational change markedly elevates proton pump output rates, ultimately maintaining body temperature through UCP1-mediated uncoupling thermogenesis [21]. Thus, the ETC is indispensable for normal physiological functions and has emerged as a prominent area of research in recent years.

### 2.2. The Production Process of the Electron Transport Chain

The ETC is a central component of cellular respiration, responsible for transferring electrons from donor molecules to final acceptors, accompanied by the establishment of a proton gradient to drive ATP synthesis. In cardiac muscle cells, more than 95% of cellular respiration is contributed by the ETC [10]. The ETC receives electrons from NADH and from succinate, with NADH acting as the initial electron donor for complex I, and succinate donating electrons to complex II (succinate dehydrogenase) where FADH_2_ functions as an internal electron carrier. These electron sources are linked to the TCA cycle and β-oxidation, respectively [22].

NADH transfers electrons to complex I (NADH-ubiquinone oxidoreductase), which transfers electrons to CoQ via iron-sulfur clusters and FMN (flavin mononucleotide), accompanied by proton pumping from the mitochondrial matrix into the intermembrane space [23]. It has been shown that complex I pumps four protons per electron transfer, establishing a transmembrane proton gradient [24].

Subsequently, CoQ acts as a mobile electron carrier, transferring electrons from complex I or complex II (succinate dehydrogenase) to complex III (cytochrome bc_1_ complex) [25]. FADH_2_ in complex II accepts electrons but does not participate in proton pumping, directing electrons only to the CoQ pool [26]. In complex III, electrons are sequentially transferred to cytochrome c via the Q-cycle mechanism, with additional proton pumping to enhance the proton gradient [27].

Next, cytochrome c acts as a soluble electron carrier, transferring electrons from complex III to complex IV (cytochrome c oxidase) [28]. Complex IV transfers electrons ultimately to molecular oxygen (O_2_) to form water molecules while pumping protons into the intermembrane space, with each pair of electron transfers accompanied by the displacement of two protons [29]. Succinate provides an alternate electron entry point into the ETC via complex II, while oxygen remains the terminal electron acceptor, with electrons flowing from succinate to O_2_.

Throughout this process, the establishment of a proton gradient drives ATP synthase (complex V) to synthesize ATP via a chemiosmotic mechanism [30]. It has been shown that each molecule of NADH generates approximately 2.5 molecules of ATP via the ETC, while FADH_2_ generates about 1.5 molecules of ATP [31].

## 3. Mitochondrial Reverse Electron Transport

As the core hub of cellular energy metabolism, the ETC not only undertakes the important task of synthesizing ATP to provide energy for various cellular life activities but also participates in the regulation of cellular redox state, apoptosis, and cellular signaling through its complex structure and fine regulatory mechanisms. However, this sophisticated system is not invulnerable. Dysfunction of the ETC may lead to insufficient ATP synthesis, affecting normal cellular function and even triggering apoptosis [32]. Additionally, abnormalities in the ETC have been closely associated with the development of various diseases, such as neurodegenerative diseases, cardiovascular diseases, and cancer [33,34,35]. In recent years, under certain pathological conditions such as hypoxia, mitochondrial diseases, and some tumors, a reverse flow of the ETC has been observed—where the direction of electron transport is opposite to that in normal physiological states. Under conditions where complex III/IV activity is compromised, the resultant CoQH_2_ accumulation reverses succinate dehydrogenase (SDH/complex II) so that it reduces fumarate to succinate, thereby re-oxidizing CoQH_2_ to CoQ and sustaining electron flow into complex I and dihydroorotate dehydrogenase (DHODH) [36]. An analogous SDH reversal is observed when high H_2_S levels inhibit complex IV: sulfide quinone oxidoreductase (SQOR)-mediated H_2_S oxidation is maintained by complex II working in reverse to regenerate CoQ, with fumarate serving as the terminal electron acceptor [37]. During I/R, succinate that accumulated via SDH during ischemia is rapidly re-oxidized upon reperfusion, driving extensive RET at complex I and ROS-mediated injury [38]. In glioblastoma stem cells, Notch2 further amplifies RET by interacting with complex I subunits, lowering the NAD^+^/NADH ratio and sustaining proliferation through SIRT1-dependent signaling; pharmacological inhibition of this RET suppresses tumor growth [39].

### 3.1. Occurrence of RET

**Figure 1:**
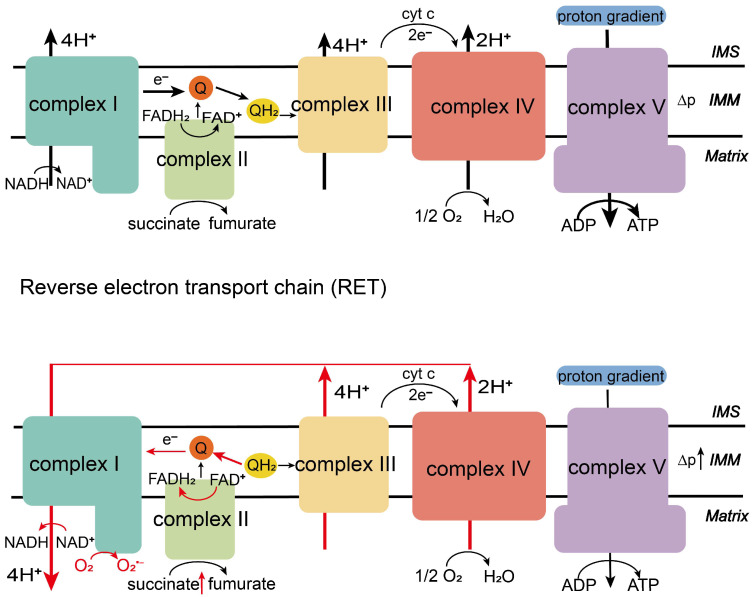
ETC (**up**): Electrons from NADH (via complex I) or succinate (via complex II) flow through the respiratory chain (complexes III–IV), driving proton pumping and ATP synthesis. RET (**down**): Under high proton motive force (Δp), accumulated succinate and CoQH_2_ drive electrons backward from complex II to complex I, generating ROS at complex I. (IMS: mitochondrial intermembrane space; IMM: inner mitochondrial membrane).

RET is a process opposite to conventional ETC function, in which electrons flow backward from complex II or the CoQ pool to complex I, reducing NAD^+^ to produce NADH and generating ROS, particularly O_2_^•−^ (Figure 1). This phenomenon was first observed in the 1960s by Chance and Hollunger, who found that adding succinate to mitochondria at high membrane potential and in a state of low ATP synthesis resulted in NAD^+^ reduction—a process sensitive to uncoupling agents, suggesting that electrons may flow in the reverse direction [8,40]. Furthermore, blocking complex III or complex IV similarly creates conditions for RET. For instance, RET is triggered when complex IV is inhibited by nitric oxide (NO) [41].

Subsequent studies have revealed that RET occurs when the CoQ pool becomes over-reduced by electrons from respiratory complex II. RET is considered an energetically uphill process requiring a proton motive force (Δp) and a highly reduced CoQ pool, usually driven by succinate accumulation through complex II activity [38]. Δp, representing the proton gradient across the inner mitochondrial membrane, makes forward electron transfer through complex I unfavorable for RET. Electron transfer follow redox potential to move, thus, accumulation of Δp drops CIII redox potential, making ubiquinone to react with substrates with higher redox potential, like complex I. According to the research by Lambert, superoxide production during RET in complex I is strongly dependent on the components of Δp [42]. Jia et al. experimentally attenuated Δp using FCCP, a protonophore uncoupler, which effectively suppressed H_2_S-induced RET and subsequent ROS generation. This observation confirmed the strict dependence of RET on mitochondrial membrane potential [43]. In vivo, when ATP synthesis is reduced and Δp accumulates, RET is more likely to occur, especially when energy demands are low or mitochondrial function is impaired. The highly reduced CoQ pool is typically driven by metabolic substrates. Succinate accumulation is particularly pronounced under ischemic conditions, with reperfusion driving RET [38].

Initially, RET was thought to exist only as an in vitro experimental phenomenon, but recent studies have confirmed its in vivo physiological and pathological significance. During macrophage activation, RET participates in regulating intracellular redox status and signaling. In I/R injury pathology, aberrant RET activation leads to excessive ROS production and exacerbates tissue damage [38].

### 3.2. Biological Functions of RET

#### 3.2.1. The Double-Edged Role of RET-ROS

ROS are a group of reactive molecules, including O_2_^•−^, H_2_O_2_, and •OH, primarily generated in the mitochondrial ETC. In the ETC, ROS are mainly produced at complex I, complex II, and complex III. RET acts as a specific mechanism in which electrons are transferred backward from the CoQ pool to complex I, generating O_2_^•−^. ROS are often regarded as metabolic by-products associated with aging and disease due to their oxidative damage to DNA, proteins, and lipids. However, recent studies show that at low levels, ROS can function as signaling molecules to regulate gene expression, cell proliferation, and apoptosis [38,44,45]. The biological function of RET-ROS signaling acts as a double-edged sword, whose divergent effects are determined by the concentration of ROS and the cellular environment, encompassing physiological homeostasis or various pathological states.

RET generates substantial ROS, particularly superoxide, at mitochondrial complex I under conditions of high membrane potential or succinate accumulation, such as during ischemia–reperfusion injury, where rapid RET-driven ROS production exceeds the scavenging capacity of enzymes like superoxide dismutase (SOD), peroxiredoxins (Prx), and glutathione peroxidase, leading to oxidative damage and cellular dysfunction [38,46]. Excessive ROS generated by RET trigger cells to establish a “first pause, then clear” two-stage buffering system. First, the increase in membrane potential caused by RET is sensed through the rate-limiting action of cytochrome c oxidase (COX). COX reduces its activity through Y304 phosphorylation mediated by kinases such as PKA or S47 phosphorylation of cyt c, thereby pausing the electron flow and reducing ROS production [47]. Subsequently, the elevated H_2_O_2_ is detected by cytochrome c peroxidase (Ccp1) in the intermembrane space of mitochondria: Ccp1 can still function as a signalling molecule in its catalytically inactive state, activating mitochondrial catalase Cta1 and inhibiting Prx, thereby enhancing H_2_O_2_ clearance [48]; simultaneously, Ccp1 signalling indirectly inhibits Sod2 activation by reducing H_2_O_2_ levels, leading to a relative increase in O_2_^•−^ levels, thereby forming a negative feedback regulation of H_2_O_2_. When Ccp1 or Cta1 is absent, H_2_O_2_ clearance is impaired, resulting in ROS accumulation and oxidative damage such as lipid peroxidation [47,48].

Jason R. et al. proposed a two-site model for superoxide production in complex I; one is the site in equilibrium with the NAD pool, presumably the flavin of the FMN moiety (site IF), and the other is the site dependent not only on NAD redox state, but also on protonmotive force and the reduction state of the Q pool, presumably a semiquinone in the Q-binding site (site IQ) [49]. Recent studies demonstrate that the IQ site of mitochondrial complex I efficiently generates ROS during both forward (FET) and RET, with sensitivity to S1QELs (site IQ electron leakage inhibitors) in both modes. Wong et al. first showed that S1QELs suppress IQ site-derived ROS by directly binding to complex I and modulating Q-site conformational changes without impairing electron flux [50]. Gibbs et al. further revealed that RET is not the exclusive condition for IQ site ROS production. During FET, the IQ site exhibits comparable ROS generation rates and maintains identical sensitivity to S1QELs, rotenone, and piericidin A as observed during RET. Using a novel rotenone challenge assay, they confirmed substantial physiological ROS production at the IQ site under FET-dominated conditions in cells, which S1QELs effectively inhibit. This finding not only explains the mechanistic basis for S1QELs’ robust ROS suppression in cellular and in vivo models but also redefines mitochondrial ROS origins by demonstrating that IQ site production is electron transport direction-independent [51]. Instead, it is governed by integrated factors including the QH_2_/Q ratio, Δp, and ΔpH.

At low levels, RET-ROS can play beneficial roles, particularly in lifespan extension, stress adaptation, and immune responses. Scialò et al. expressed yeast NADH dehydrogenase (NDI1) in Drosophila, which over-reduced the CoQ pool and increased RET-produced mtROS, extending the lifespan of Drosophila. When mitochondria-targeted catalase (mtCAT) expression restored normal H_2_O_2_ levels, the lifespan of Drosophila returned to that of the control group, confirming that mtROS is key to NDI1-mediated lifespan extension and highlighting the critical role of RET-ROS in signaling [45]. Subsequently, they observed increased RET-ROS in Drosophila exposed to heat stress at 32 °C for 3 h, activating the transcriptional stress response, including stress-related gene expression and enhancing survival. In contrast, inhibiting RET-ROS reduced the stress response and significantly shortened survival under heat stress [52].

In cellular immune signaling, Mills et al. found that in macrophages, ROS generated by RET triggered by succinate oxidation are critical for IL-1β activation and production, enhancing host defense during bacterial infection [53]. Follow-up experiments have also supported the important role of RET-generated mtROS in NLRP3 inflammasome activation—a key mitochondria-derived signal regulating cytokine production in macrophages [54]. RET-ROS also play a significant role in metabolic regulation, influencing various metabolic pathways. For example, Ni et al. demonstrated that in ischemic stroke, RET-ROS regulates astrocytic mitochondrial function, promoting mitochondrial transfer to neurons and attenuating oxidative damage [55].

Under pathological conditions, RET-ROS may cause significant harm. Chouchani et al. showed that in I/R injury, such as after a heart attack or stroke, complex II rapidly oxidizes accumulated succinate, reduces the CoQ pool, and drives RET at complex I, generating a ROS burst that leads to oxidative damage and cell death [38]. In ischemic stroke, the RET-ROS burst causes mitochondrial dysfunction and neuronal death [9]. Regarding aging, Rimal et al. demonstrated that senescent Drosophila loses the ability to regulate RET-ROS, and mitochondria produce persistently high ROS levels, correlating with aging-related phenotypes such as shortened lifespan and decreased health span. RET inhibition prolongs lifespan and improves crawling activity and mitochondrial function [56].

In AD models, RET activation increased ROS and decreased the NAD^+^/NADH ratio, while RET inhibition extended lifespan and improved memory and motor function in Drosophila and mouse AD models [56]. Roca et al. found in tuberculosis that TNF induces pathological ROS via RET, exacerbating inflammation and tissue damage [57]. As a pathogenic hub in multiple diseases, the mitochondrial complex I has been implicated in I/R injury. Yin et al. provided the most direct in vivo evidence to date using ND6-P25L knockin mice: a single mtDNA point mutation specifically abolishes complex I’s ability to catalyze RET-dependent ROS generation while preserving NADH oxidation and ATP production. In a myocardial I/R model, this mutation prevented succinate-driven ROS bursts and reduced infarct size by approximately 80% compared to wild-type controls. This study genetically establishes the RET-ROS pathway as the central mechanism driving cardiac I/R injury and identifies complex I-RET as a precise therapeutic target [58]. Additionally, studies have shown that in experimental autoimmune encephalomyelitis (EAE) models, microglia generate mtROS through RET driven by mitochondrial complex I, sustaining chronic neuroinflammation and causing neurotoxicity. However, ND6-P25L mutant mice significantly alleviate disease in EAE by blocking RET, confirming that inhibiting RET is a viable therapeutic strategy [59].

RET-ROS exhibits multiple facets in cell signaling and metabolic regulation, demonstrating beneficial effects at low levels for stress adaptation but potentially damaging effects at high levels. The dual role of RET-ROS remains controversial, particularly regarding the balance between its benefits and harms. Differences in experimental conditions, such as model organisms and ROS levels, may lead to varying results, necessitating further clarification. Additionally, the metabolic regulatory role of RET-ROS differs across cell types, emphasizing the need for systemic studies to understand its function. Unraveling the mechanisms underlying its dual role is essential for developing therapeutic strategies targeting ROS.

#### 3.2.2. Effects of RET on Energy Metabolism and Physiological Functions

As previously described, under physiological conditions, the ETC operates in the forward direction, transferring electrons from NADH and FADH_2_ to molecular oxygen via complexes I–IV to establish Δp across the inner mitochondrial membrane. This Δp, primarily driven by mitochondrial membrane potential (ΔΨm), enables ATP synthase (complex V) to generate ATP through OXPHOS. ATP serves as the primary energy currency of the cell, and this standardized process ensures metabolic homeostasis, supporting processes such as ion transport, biosynthesis, and muscle contraction.

In contrast, RET typically occurs under conditions of elevated succinate levels or altered metabolic states, where electrons flow backward from CoQH_2_ to complex I, driven by high ΔΨm and a reduced CoQ pool. Once considered a by-product of mitochondrial dysfunction, RET has emerged as a key regulator of energy metabolism. Unlike the ETC, which prioritizes ATP generation, RET redirects energy fluxes to influence metabolic pathways and cell fate in ways that extend beyond simple bioenergetics.

One of the most significant metabolic consequences of RET is its modulation of proton motive force and its impact on ATP synthase activity. Casey et al. demonstrated in lipopolysaccharide (LPS) -stimulated bone marrow-derived macrophages that succinate-driven RET at complex I elevated ΔΨm without a proportional increase in ATP production. LPS-induced metabolic reprogramming shifted ATP synthesis from OXPHOS to glycolysis, reducing F_0_F_1_-ATP synthase activity and maintaining high ΔΨm. This suggests that RET serves as an energy-dissipating mechanism, potentially protecting mitochondria from hyperpolarization-induced stress while redirecting metabolic intermediates to alternative pathways [54].

Mills et al. observed in LPS-stimulated mouse macrophages that succinate-driven RET enhanced the reverse activity of complex I, increasing mitochondrial membrane potential [53]. Prag et al. found in a murine cardiac I/R model that rapid succinate oxidation during early reperfusion drove RET, a process accompanied by energy dissipation rather than ATP synthesis [60]. In adipose tissue thermogenesis, succinate oxidation by SDH activates thermogenic respiration, while in cardiac I/R, RET may similarly influence energy utilization efficiency. Malonate, a competitive SDH inhibitor, reduces RET and energy dissipation, decreasing myocardial infarct size suggesting that RET influences energy utilization efficiency through Δp dissipation [61]. Scialò et al. found in Drosophila mitochondria that RET, driven by high ΔΨm, reduced ATP synthesis efficiency, redirecting energy to support metabolic adaptations. Knockdown of the complex I subunit NDUFS3 reversed this effect [52]. These studies collectively indicate that RET preferentially dissipates energy rather than synthesizing ATP during metabolic stress.

RET also reduces the intracellular NAD^+^/NADH ratio, which is critical for metabolic pathways. A high NADH/NAD^+^ ratio inhibits key enzymes, impairing glycolysis and the TCA cycle, thereby reducing ATP production. Recent studies suggest that RET activation during aging depletes NAD^+^, potentially contributing to energy metabolism dysfunction [56]. Under certain conditions, RET may act adaptively to generate NADH, maintaining redox homeostasis or supporting biosynthesis. In I/R, RET may promote cell survival by generating NADH at the cost of reduced ATP production [38]. This dual role underscores the complexity of RET in metabolic adaptation.

Over the past decade, studies have functionally linked RET to ROS generation and NAD^+^/NADH-mediated signaling by expressing prokaryotic/yeast alternative oxidase (AOX) in mammalian systems lacking complex I activity, establishing RET as a pivotal regulator of mitochondrial-nuclear crosstalk. In the AOX-expressing cardiac mitochondrial model developed by Robb et al., Ciona intestinalis AOX continuously oxidizes CoQH_2_ to suppress succinate-driven RET-ROS. Pharmacological inhibition of AOX with N-propyl gallate induces transient RET-ROS rebound, providing the evidence that complex I’s reverse electron flow acts as a molecular switch for ROS signaling [62]. Szibor et al. expressed AOX in I/R model, finding that AOX maintained electron flux and significantly reduced post-reoxygenation mitochondrial ROS bursts caused by RET. Despite reducing ROS production, AOX failed to confer acute or long-term cardiac protection. Instead, it inhibited post-ischaemic Anp upregulation, leading to increased collagen deposition and worsened long-term contractile function [63]. Ojha et al. revealed a ROS-independent mechanism in glioblastoma (GBM) stem cells: Notch2 physically binds complex I’s Fe-S cluster subunits (NDUFV1/NDUFS2/NDUFS3) to promote RET, which maintains tumorigenicity primarily through NAD^+^/NADH reduction. Both AOX expression and pharmacological RET inhibition normalized NAD^+^/NADH ratios and suppressed tumor sphere formation [39]. Collectively, these studies demonstrate that during complex I dysfunction, exogenous AOX intercepts RET to disrupt mitochondrial signaling cascades. This work positions RET as a molecular switch coordinating electron flow with transcriptional reprogramming, inflammatory responses, and stem cell fate determination.

#### 3.2.3. Effects of RET on Unnatural Organismal Death

Mounting evidence indicates that RET is not only involved in normal physiological functions but also closely associated with abnormal cell or organism death, including programmed cell death (e.g., pyroptosis) and newly discovered pathways like ferroptosis and PANoptosis. Mitochondrial dysfunction, ΔΨm abnormalities, and alterations in key intracellular substances often accompany the triggering of these death forms.

RET plays a significant role in necrosis. As a key host resistance factor against tuberculosis, TNF is a critical host resistance factor against tuberculosis, but excess TNF in infected macrophages increases mtROS production via RET at complex I, triggering a signaling cascade that leads to pathological necrosis of Mycobacterium tuberculosis-infected macrophages [57].

Pyroptosis, a programmed cell death dependent on inflammasome activation, involves caspase activation and Gasdermin D (GSDMD) cleavage, ultimately perforating cell membranes and releasing inflammatory factors. RET can indirectly or directly trigger pyroptosis by altering the mitochondrial intracellular environment. RET-mediated mtROS not only directly induce pyroptosis but may also promote cell death by activating the RIPK1/RIPK3/MLKL pathway [64]. Additionally, RET enhances mtDNA release. Oxidized mtDNA (ox-mtDNA) activates the NLRP3 inflammasome, a key pyroptosis mediator, leading to GSDMD cleavage and cell lysis [65]. Anti-RET reagents significantly reduce mtROS and block NLRP3 activation, inhibiting pyroptosis.

Ferroptosis, an iron- and lipid peroxidation-dependent cell death form, has recently garnered attention for its connection to RET. During I/R, succinate accumulated during ischemia is rapidly oxidized by mitochondrial SDH upon reperfusion, driving RET at complex I of the ETC and resulting in substantial ROS production. These ROS subsequently trigger lipid peroxidation, ultimately inducing neuronal ferroptosis; conversely, the TP53-induced glycolysis and apoptosis regulator (TIGAR) translocates to mitochondria under hypoxic conditions, where it interacts with the SDHA subunit of SDH and modulates its post-translational modifications—promoting acetylation while inhibiting succinylation—thereby reducing SDH activity. This suppression of SDH activity blocks RET, attenuates ROS generation and lipid peroxidation accumulation, and ultimately inhibits ferroptosis [66]. PANoptosis, a novel programmed cell death combining pyroptosis, apoptosis, and necroptosis features, is mediated by the PANoptosome complex and is highly pro-inflammatory [67]. RET-mediated mtROS induce mtDNA oxidation, promoting PANoptosome assembly and activation of all three death pathways. Conversely, anti-RET reagents inhibit PANoptosis by blocking mtROS and mtDNA oxidation [68].

### 3.3. Relationship of RET to Disease Occurrence and Treatment

#### 3.3.1. RET and Ischemia–Reperfusion (I/R)

RET plays a key role in I/R, and most studies on RET have been conducted using I/R injury models. I/R was first proposed by Jennings et al. in 1960 using a canine heart coronary ligation model [69]. It refers to the phenomenon where the restoration of blood flow to ischemic tissues results in more severe cellular damage and dysfunction. This process is closely associated with mitochondrial dysfunction, particularly the ROS burst triggered by RET.

**Figure 2:**
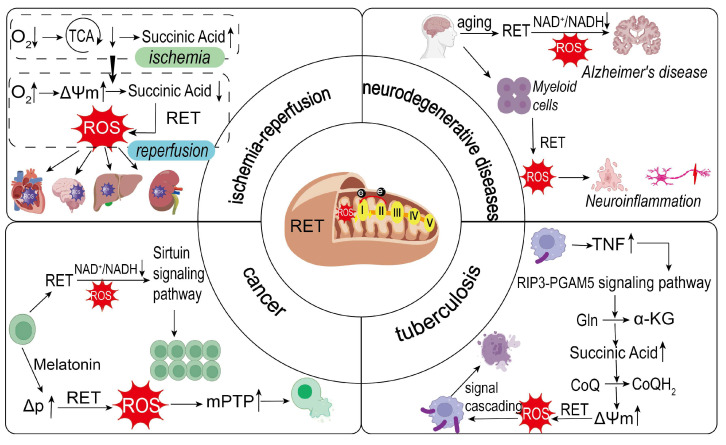
RET triggers the generation of ROS, which in turn affects the progression of various diseases such as ischemia–reperfusion injury, neurodegenerative diseases, cancer and tuberculosis.

During I/R, the ischemic phase leads to insufficient oxygen supply, impaired mitochondrial tricarboxylic acid cycle metabolism, and significant accumulation of metabolites such as succinate. Studies have shown that succinate can accumulate to several times its normal level in ischemic myocardium, providing a key substrate for RET during reperfusion [70]. Upon reperfusion, the rapid recovery of oxygen reestablishes a high ΔΨm, which drives the reverse flow of electrons, facilitating the rapid oxidation of succinate and inducing RET. This leads to a sudden increase in ROS levels (Figure 2).

The rapid oxidation of succinate is a major source of ROS generation in the early reperfusion phase, and RET is a key mechanism underlying this process. In myocardial I/R models, succinate accumulation during ischemia is necessary for RET, and its rapid metabolism post-reperfusion drives ROS generation, causing cardiomyocyte injury [38]. RET-mediated ROS generation is closely linked to oxidative stress and tissue injury. Inhibition of SDH significantly reduces RET-mediated ROS production, thereby attenuating reperfusion injury. Research indicates that RET-mediated ROS generation accounts for over 90% of ROS in the early reperfusion phase. Although this value may be overestimated due to other contributing factors, RET remains a major driver of I/R injury [71].

As the core site of RET, the structure and regulatory mechanisms of complex I directly influence ROS generation efficiency. During ischemia, complex I transitions from the Active (A) to the Deactivated (D) state, and reverts to the A state during reperfusion, potentially enhancing RET activity [72]. Additionally, within complex I, the mitochondrially encoded subunit ND2 (MT-ND2) and flavin mononucleotide-binding subunits are most susceptible to oxidative damage under I/R conditions, as they constitute the main superoxide generation site during RET [73]. These molecular mechanisms may provide a theoretical basis for interventions targeting complex I.

RET contributes to I/R injury in various tissues. For example, succinate accumulation in ischemic myocardium drives RET, leading to ROS bursts and cardiomyocyte necrosis [60]. SDH inhibitors such as dimethylmalate (DMM) significantly reduce myocardial infarct size [74]. Lowering membrane potential or using uncoupling agents may also attenuate myocardial injury by inhibiting RET [60]. Animal models have demonstrated that complex I or SDH inhibitors reduce post-stroke brain damage, highlighting RET’s potential as a therapeutic target [75]. Mitochondria-targeted antioxidants like MitoQ have shown protective effects by reducing RET-associated ROS generation in mouse models [76].

I/R injury is a major cause of acute kidney injury (AKI), for which effective treatments remain lacking. Succinate accumulation during ischemia and its subsequent oxidation during reperfusion result in excessive ROS production and severe kidney damage. Studies indicate that succinate accumulation and RET-mediated ROS generation contribute to renal tubular cell injury [77]. Antioxidants such as Mito-TEMPO significantly improve renal function in cisplatin-induced AKI mice by scavenging mitochondrial superoxide in renal tubular cells [78] (Figure 2). Additionally, research has demonstrated that H_2_S delivered via NaHS donors exerts neuroprotective effects by suppressing the NLRP3/caspase-1/GSDMD pathway. This inhibition attenuates I/R-induced neuroinflammation and necroptosis in both cerebral cortical and retinal tissues, suggesting H_2_S donors as promising therapeutic candidates for ischemic stroke [79].

#### 3.3.2. RET and Neurodegenerative Diseases

Neurodegenerative diseases are characterized by progressive neuronal dysfunction and structural loss, leading to cognitive and motor impairments [80]. RET may exacerbate disease progression by triggering oxidative stress and disrupting energy metabolism in pathological states (Figure 2). RET and the ROS it produces are activated in neurodegenerative diseases, leading to ROS accumulation and a decrease in the NAD^+^/NADH ratio, which in turn promotes disease progression.

AD, the most common form of dementia in the elderly, is characterized by amyloid plaques and neurofibrillary tangles. Aberrant RET activation has been observed in AD patient brain tissues and animal models, leading to increased ROS and reduced NAD^+^/NADH ratios. Rimal et al. discovered that RET was progressively enhanced in AD patients’ brains and two transgenic mouse models, showing positive correlation with disease severity. This enhancement was associated with decreased NAD^+^/NADH ratios and elevated ROS levels. Mechanistically, APP-C99 binds to complex I subunits NDUFS3 and NDUFV1, weakening the NDUFS3-NDUFV1 interaction while strengthening NDUFS3-NDUFV2 binding. The specific RET inhibitor CPT suppresses RET by binding to complex I and restoring normal NDUFS3-NDUFV1 interactions [81]. Aging, a major AD risk factor, is associated with RET activation. Rimal et al. demonstrated that in an aging Drosophila model, RET at mitochondrial complex I becomes activated, resulting in elevated ROS production and decreased NAD^+^/NADH ratios. Pharmacological inhibition of RET (using CPT or rotenone) or genetic suppression (via knockdown of complex I subunits NDUFS3 and NDUFS2) significantly reduced ROS generation and restored NAD^+^/NADH homeostasis. These interventions not only extended lifespan in wild-type flies but also attenuated abnormal protein translation and aggregation while providing neuroprotection [56]. These findings suggest RET as a key driver in AD pathology and a potential therapeutic target.

Neuroinflammation is a hallmark of neurodegenerative diseases like multiple sclerosis (MS). Recent studies reveal that RET in myeloid cells drives ROS production via complexes I and II, sustaining myeloid cell activation and exacerbating neuroinflammation. In animal models, RET blockade significantly reduces central nervous system neurotoxicity and improves functional outcomes [82]. This implicates RET as a pathological driver and therapeutic target in neuroinflammation-related diseases. In MS, RET-mediated ROS may exacerbate axonal damage and neuronal death, suggesting that RET inhibition could slow disease progression.

#### 3.3.3. RET and Cancer

In the ETC, Complexes I and II transfer electrons to CoQ to generate CoQH_2_, while complex III reoxidizes ubiquinol back to ubiquinone. This cyclic process maintains the cellular ubiquinone pool, which supports essential metabolic functions including pyrimidine biosynthesis and TCA cycle activity. Notably, this mechanism is crucial for sustaining tumor growth [83]. In cancer, RET-generated ROS plays a dual role in tumor cell fate. RET-driven ROS supports survival and proliferation, particularly in cancer stem cells (CSCs). Ojha et al. demonstrated that RET at complex I exhibits heightened activity in brain tumor stem cells. RET-mediated reduction of NAD^+^ to NADH decreases the NAD^+^/NADH ratio, consequently suppressing the NAD^+^-dependent SIRT1 signaling pathway. This metabolic reprogramming drives cancer cell proliferation and survival. The Notch pathway regulates this process through non-canonical mitochondrial translocation of Notch2, which directly interacts with iron-sulfur cluster-containing complex I subunits (NDUFV1 and NDUFS3) to modulate RET activity. Pharmacological RET inhibition (e.g., using CPT) restores NAD^+^/NADH homeostasis, suppresses tumor growth, and represents a novel therapeutic strategy for cancer treatment [39].

Excessive ROS from RET can induce oxidative stress, leading to cancer cell death (Figure 2). For example, in head and neck cancer, melatonin reprograms cellular metabolism to over-reduce CoQ and elevate mitochondrial membrane potential; these conditions initiate RET through complex I, producing a burst of ROS. The ensuing oxidative insult opens the mitochondrial permeability transition pore (mPTP)—a Ca^2+^ and ROS-sensitive, high-conductance channel spanning the inner mitochondrial membrane whose opening depolarizes the organelle, uncouples oxidative phosphorylation, and commits the cell to apoptosis. Melatonin’s antitumor effect was diminished without RET, confirming RET-mediated ROS as its core mechanism [84].

ROS exhibit dual roles in tumor biology: moderate levels promote growth via signaling, while excess ROS triggers oxidative damage to lipids, proteins, and DNA, inducing cell death. Tumour cells maintain RET-ROS at a level that promotes proliferation by upregulating the glutathione system (GSH/GPx/GR, etc.) and its synergistic action with the thioredoxin system and NADPH regeneration pathway [35]. However, therapeutic interventions can disrupt redox homeostasis by increasing ROS or inhibiting antioxidants, inducing cell death [85].

#### 3.3.4. RET and Tuberculosis

Tuberculosis (TB), caused by Mycobacterium tuberculosis (Mtb), involves bacterial survival within macrophages by manipulating host metabolism and immune responses [86]. In TB, RET accompanied by high mitochondrial ROS production is a key mechanism of Mtb-induced pathology (Figure 2). TNF is crucial for TB immune defense, but excess TNF exacerbates pathology. Roca et al. found that in Mtb-infected macrophages, excess TNF activates glutamine uptake via the RIP3-PGAM5 pathway, enhancing glutamine catabolism to α-ketoglutarate and increasing succinate levels. Succinate oxidation at complex II reduces CoQ to CoQH_2_ and elevates ΔΨm, driving RET at complex I and generating mROS bursts. These mROS activate mitochondria-lysosome-endoplasmic reticulum signaling, promoting macrophage necrosis and Mtb release [57]. While moderate TNF enhances bactericidal activity via mROS, excess TNF induces necrosis via the RIP1-RIP3 pathway [87].

Succinate is central to RET. Tannahill et al. showed succinate stabilizes HIF-1α, promoting pro-inflammatory responses [88]. In TB, Mtb infection upregulates macrophage succinate levels, driving RET and mROS production [57]. Mills et al. demonstrated that succinate oxidation depends on complex II activity, with its inhibition significantly reducing mROS [53].

RET plays a pivotal role in TB pathology by regulating mROS. TNF, succinate, and Mtb virulence factors synergistically drive RET, inducing macrophage necrosis and bacterial proliferation. Further research is needed to elucidate RET’s complex mechanisms in TB to optimize therapies.

#### 3.3.5. Treatment of RET-Related Diseases

For RET-associated diseases, therapeutic approaches include inhibiting complexes and metabolites to mitigate RET’s effects.

Among these strategies, reducing succinate accumulation has emerged as a particularly promising avenue. In I/R injury, where RET drives cellular damage in myocardial infarction and stroke, RET inhibition is crucial for cardiovascular protection. SDH inhibitors like malonate reduce succinate oxidation, decreasing RET and ROS during reperfusion. Malonate has shown efficacy in reducing infarct size and preventing post-MI heart failure in mice, though its mechanisms require further study [89,90]. Baicalin inhibits SDH-mediated oxidative stress in early reperfusion, suggesting potential for acute ischemic stroke treatment [91]. The apoptosis regulator TIGAR reduces neuronal ferroptosis by inhibiting SDH in cerebral ischemia [66]. Thus, targeting succinate metabolism during reperfusion could complement I/R treatment.

ROS also play a central role in RET-driven pathogenesis, with their overproduction triggering a cascade of damaging cellular events. ROS generated during RET act as the proximal trigger of mitochondrial injury: they open the mPTP, collapse ΔΨm, and release cytochrome c, thereby activating the apoptotic cascade [92,93]. Therapeutic modulation of ROS production or clearance can ameliorate RET-driven pathology. In I/R injury, xanthine oxidase (XO) serves as a major ROS source. Febuxostat, a selective XO inhibitor, attenuates ROS generation by blocking XO activity. As a lipophilic antioxidant, vitamin E directly scavenges free radicals during the reperfusion-phase ROS burst and reduces ETC lipid peroxidation, collectively mitigating I/R injury [94]. MitoQ, a mitochondria-targeted CoQ mimetic, alleviates AD-associated neuroinflammation through ROS neutralization [95] and suppresses cancer cell motility and proliferation in breast and pancreatic malignancies by decreasing intracellular ROS levels [96,97]. α-Lipoic acid (ALA), the lipoyl-cofactor of pyruvate dehydrogenase (PDH) and α-ketoglutarate dehydrogenase (KGDH), is synthesized by the [4Fe-4S] enzyme lipoic acid synthase (LIAS). During myocardial I/R, ROS oxidatively disrupt LIAS, leading to reduced lipoylation and decreased TCA cycle efficiency. Combined treatment with MitoQ and ALA exerts a dual protective mechanism. MitoQ curtails ROS production, thereby preserving LIAS integrity and de novo ALA synthesis. Meanwhile, exogenous ALA directly relipoylates PDH and KGDH, sustaining the generation of NADH and FADH_2_. This coordinated approach results in reduced mitochondrial ROS and malondialdehyde (MDA) levels, restored SOD and glutathione peroxidase (GPX) activities, and prevention of ΔΨm collapse. Furthermore, it shifts mitochondrial dynamics toward fusion (evidenced by upregulation of Mfn1, Mfn2, and Foxo1) and suppresses fission (indicated by downregulation of Drp1 and Fis1). Consequently, these changes lead to markedly smaller infarct sizes and improved cardiac function, as reported in previous studies [98,99].

Targeting complex I—an integral component of the ETC—also offers therapeutic potential for RET-related diseases. Metformin, a T2D treatment, inhibits the NDUFB4 subunit of complex I, reducing ROS and tumorigenesis [100,101]. In AD, CPT1C regulates ROS levels, reducing oxidative stress, apoptosis, and AD marker protein deposition [102]. CPT1C overexpression enhances mitochondrial function, reduces ROS, and reverses cellular senescence [103]. Rotenone inhibits complex I, limiting ATP synthesis, generating excess ROS, and triggering apoptosis in cancer cells, though its toxicity (e.g., bone marrow atrophy, bone growth suppression, and liver metabolic disorders) limits clinical use [104].

Additionally, modulating NAD^+^—a redox-sensitive coenzyme central to energy metabolism—provides another viable strategy for treating RET-related conditions. Nicotinamide mononucleotide (NMN) increases cardiac NAD^+^, restoring the NAD^+^/NADH ratio and reducing RET-associated ROS [105]. NMN also improves neurodegenerative diseases, such as reducing ROS and enhancing cognition in AD mice [106]. Nicotinamide riboside (NR) similarly improves I/R outcomes [107].

## 4. Conclusions

Mitochondrial RET has emerged as a fundamental regulatory mechanism in cellular bioenergetics, representing a critical interface between metabolic homeostasis and pathological processes. Our comprehensive analysis reveals that RET functions as a dynamic sensor of cellular redox status, with its biological outcomes being exquisitely dependent on the cellular context, degree of activation, and tissue-specific metabolic demands. Under physiological conditions, the controlled generation of RET-ROS serves as an essential component of cellular signaling networks, participating in immune system activation, hypoxic adaptation, and metabolic reprogramming. ROS acts as a bridge linking RET and disease, activating key stress response pathways and maintaining redox homeostasis.

However, the pathological consequences of RET dysregulation are particularly striking in several major disease states. In I/R injury, the abrupt restoration of oxygen following ischemic periods triggers uncontrolled RET activation, leading to destructive ROS bursts that account for up to 90% of total ROS production during early reperfusion. This phenomenon has been mechanistically linked to the rapid oxidation of accumulated succinate, creating a highly reduced CoQ pool that drives reverse electron flow through complex I. Similar pathological RET activation patterns have been identified in neurodegenerative diseases, where chronic RET-mediated oxidative stress contributes to neuronal dysfunction and death. The discovery that RET inhibition can ameliorate disease phenotypes in Alzheimer’s models while extending lifespan in experimental organisms underscores its potential as a therapeutic target.

The dual nature of RET presents both opportunities and challenges for therapeutic intervention. Our analysis of current strategies reveals several promising approaches: targeted suppression of RET initiation through SDH inhibition or membrane potential modulation; selective scavenging of RET-derived ROS using mitochondrial-targeted antioxidants; and metabolic preconditioning to prevent pathological RET activation. Particularly noteworthy is the development of novel compounds like DMM and site-specific complex I inhibitors that can discriminate between physiological and pathological RET. However, significant hurdles remain in achieving tissue-specific targeting and avoiding interference with essential ROS signaling functions.

Looking forward, several critical research directions demand attention. First, the molecular architecture of RET regulation requires deeper characterization, particularly the post-translational modifications of complex I that govern its propensity for reverse electron flow. Second, the development of advanced imaging modalities for real-time RET monitoring in living systems will be essential for understanding its dynamic regulation. Third, the tissue-specific metabolic consequences of RET modulation need systematic evaluation, as emerging evidence suggests significant variation in RET sensitivity across different cell types.

From a translational perspective, the most promising near-term applications appear to be in ischemia–reperfusion injury and neurodegenerative diseases, where proof-of-concept studies have demonstrated significant protection through RET modulation. The potential extension of these approaches to cancer therapy, particularly in targeting cancer stem cell metabolism, represents an exciting frontier. However, the development of clinically viable RET modulators will require careful optimization of pharmacokinetic properties and tissue selectivity.

In conclusion, RET represents a paradigm-shifting concept in mitochondrial biology, revealing how a fundamental bioenergetic process can be co-opted for signaling purposes while retaining the potential for pathological consequences when dysregulated. As our understanding of RET’s molecular mechanisms and physiological roles continues to deepen, it is becoming increasingly clear that targeted modulation of this pathway holds substantial promise for treating some of the most challenging human diseases. Future research should focus on bridging the gap between mechanistic understanding and clinical application, with particular emphasis on developing precision therapies that can selectively modulate RET in disease-affected tissues while preserving its essential physiological functions.

## Data Availability

Not applicable.
